# P-1561. Autoantibodies to Interferon-γ and Susceptibility to Mycobacterial and other Opportunistic Intracellular Pathogens

**DOI:** 10.1093/ofid/ofaf695.1741

**Published:** 2026-01-11

**Authors:** Rini Bandyopadhyay, Debasree Kundu, Mithun M George, V Nagaraj, George M Varghese

**Affiliations:** Christian Medical College, Vellore, Vellore, Tamil Nadu, India; Christian Medical College, Vellore, Vellore, Tamil Nadu, India; Christian Medical College, Vellore, Vellore, Tamil Nadu, India; Christian Medical College, Vellore, Vellore, Tamil Nadu, India; Christian Medical College, Vellore, Tamil Nadu, India

## Abstract

**Background:**

Autoantibodies against interferon-γ (IFN-γ) are increasingly associated with severe disseminated opportunistic infections. IFN-γ is a potent anti-bacterial and immunomodulatory cytokine playing a major role in controlling intracellular infections. Adult-onset immunodeficiency associated with life-threatening intracellular infections is characterized by the presence of anti-IFN-γ-autoantibodies (Abs).

**Table 1:**
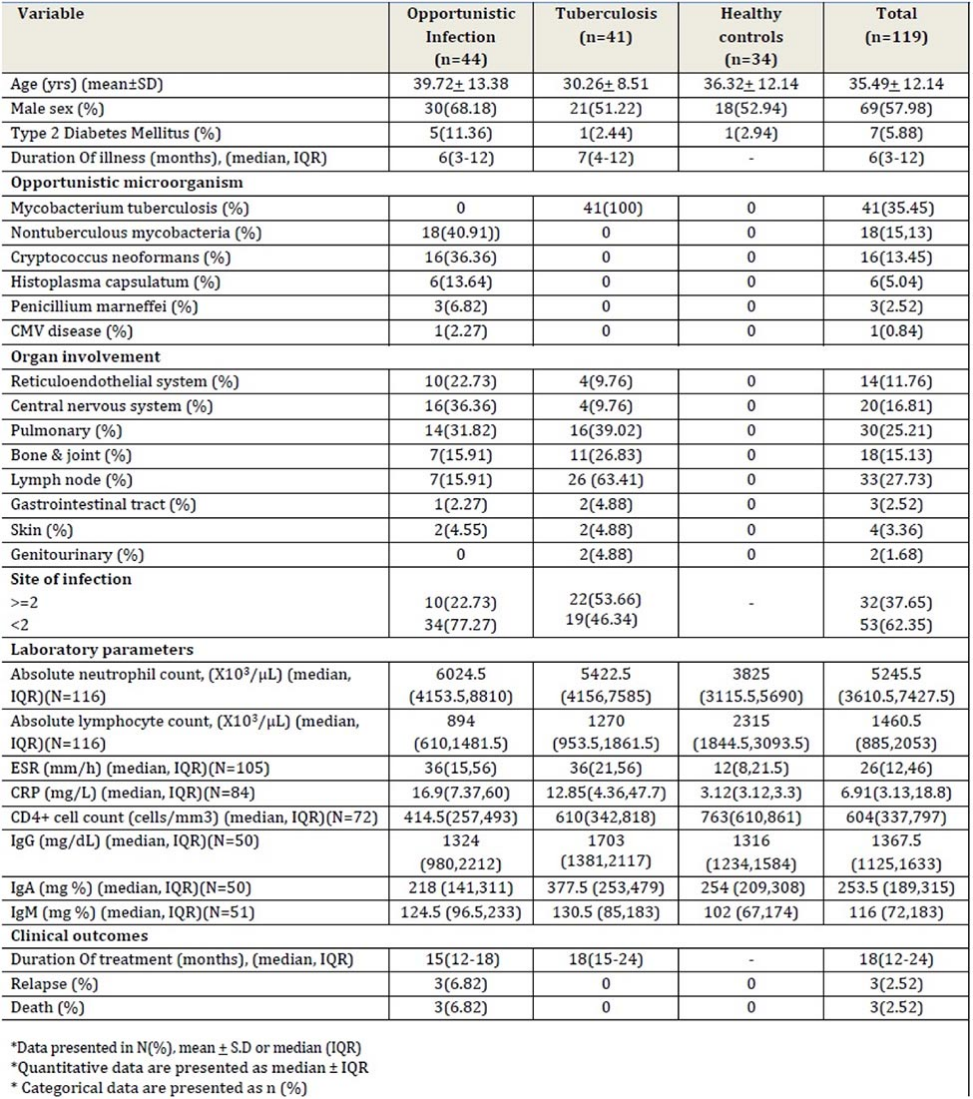
Patient characteristics

**Table 2:**
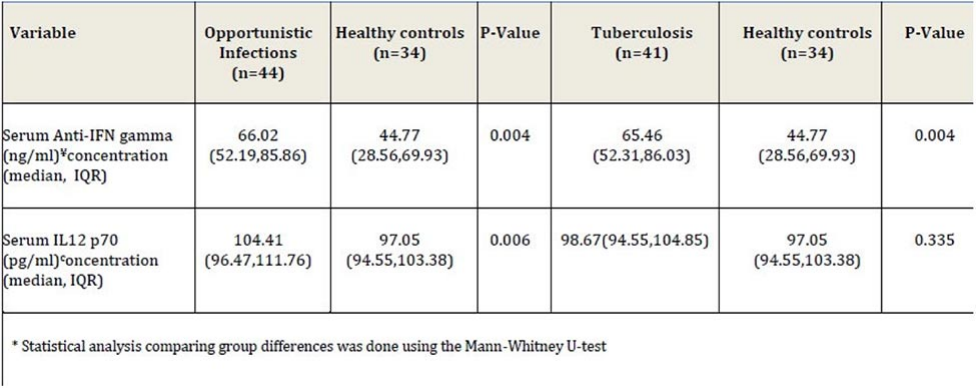
Comparison of serum anti-IFN gamma and IL12 p70 concentrations between opportunistic Infections, tuberculosis, and healthy control groups

**Methods:**

In this observational study, data and sera from 119 individuals with intracellular opportunistic infections and healthy controls were collected and analyzed. Anti-IFNγ-autoAbs and IL12p70 concentrations in serum were measured using enzyme-linked immunosorbent assay (ELISA). Median anti-IFNγ-autoAbs and serum IL12p70 levels were compared with healthy controls. Mann-Whitney U-test was used to compare group differences.

**Figure: 1:**
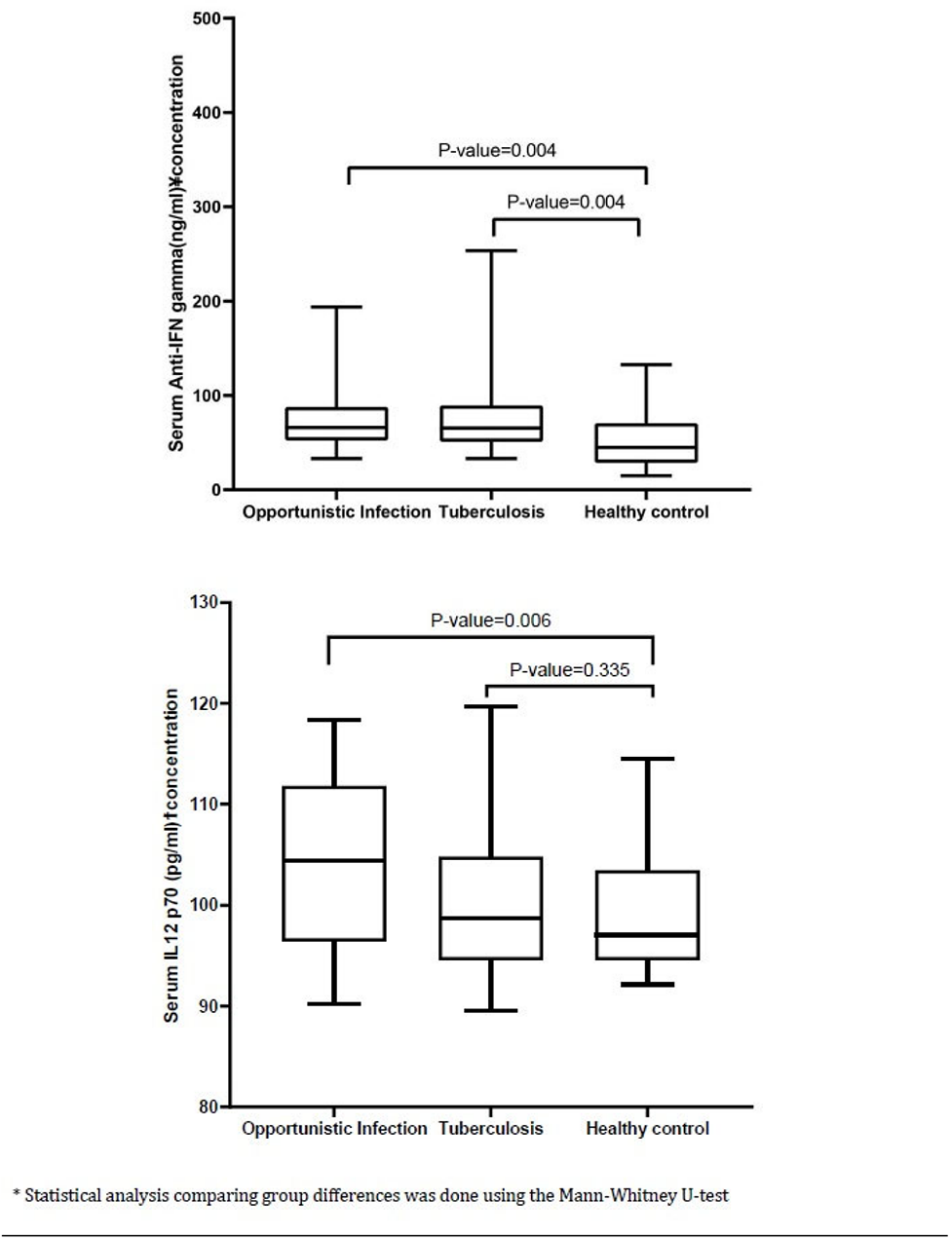
Serum anti-IFN gamma and IL12 p70 concentrations among opportunistic infections, tuberculosis, and healthy control groups

**Results:**

119 patients were enrolled in three groups: 44 patients with unusual severe disseminated opportunistic intracellular infections (group 1); 41 patients with slowly resolving tuberculosis infection (group 2); and 34 healthy controls (group 3). Disseminated non-tuberculous mycobacterial infection was the most common opportunistic infection identified 18 (40.9%) in group 1, followed by cryptococcal infections 16 (36.4%) and disseminated histoplasmosis 6 (13.6%). Median concentration of anti-IFNγ-autoAbs in group 1 was 66.02 ng/ml (IQR, 52.19, 85.86) while in group 2 and group 3 were 65.46 ng/ml (IQR, 52.31, 86.03) and 44.77 ng/ml (IQR, 28.56, 69.93). Median duration of treatment in group 1 was 15 months (IQR, 12, 18) and in group 2 was 18 months (IQR, 15, 24).

Serum concentrations of anti-IFNγ-autoAbs in group 1 and 2 exceeded the median concentrations in healthy controls by two-fold (P=0.004). Serum IL12p70 concentrations was significantly higher in group 1 (P=0.006) while in group 2, it did not differ significantly (P=0.34) as compared to healthy controls. Relapse occurred among 3 (6.8%) patients in group 1 and 3 (6.8%) patients in group 1 succumbed during the study period.

**Conclusion:**

Anticytokine autoantibodies may cause acquired susceptibility to infection and their detection impacts clinical management. Reinfection, relapse or persistent infections are common requiring close long-term surveillance.

**Disclosures:**

All Authors: No reported disclosures

